# Altered relationship between anandamide and glutamate in circulation after 30 min of arm cycling: A comparison of chronic pain subject with healthy controls

**DOI:** 10.1177/1744806919898360

**Published:** 2019-12-30

**Authors:** Niclas Stensson, Anna Grimby-Ekman

**Affiliations:** 1 Pain and Rehabilitation Centre, Department of Medical and Health Sciences, Linköping University, Linköping, Sweden; 2 Health Metrics, Department of Medicine, School of Public Health and Community Medicine, Gothenburg University, Gothenburg, Sweden

**Keywords:** Chronic pain, endocannabinoids, anandamide, glutamate, physical activity

## Abstract

The insufficient knowledge of biochemical mechanisms behind the emergence and maintenance of chronic musculoskeletal pain conditions constrains the development of diagnostic and therapeutic tools for clinical use. However, physical activity and exercise may improve pain severity and physical function during chronic pain conditions. Nevertheless, the biochemical consequences of physical activity and exercise in chronic pain need to be elucidated to increase the precision of this therapeutic tool in chronic pain treatment. The endocannabinoid system has been suggested to play an important role in exercise-induced reward and pain inhibition. Moreover, glutamatergic signalling has been suggested as an important factor for sensation and transmission of pain. In addition, a link has been established between the endocannabinoid system and glutamatergic pathways. This study examines the effect of dynamic load arm cycling (30 min) on levels of lipid mediators related to the endocannabinoid system and glutamate in plasma of chronic pain subjects and pain-free controls. Pain assessments and plasma levels of arachidonoylethanolamide (anandamide), 2-aracidonoylglycerol, oleoylethanolamide, palmitoylethanolamide, stearoylethanolamide and glutamate from 21 subjects with chronic neck pain (chronic pain group) and 11 healthy controls were analysed pre and post intervention of dynamic load arm cycling. Pain intensity was significantly different between groups pre and post exercise. Post exercise, anandamide levels were significantly decreased in health controls but not in the chronic pain group. A strong positive correlation existed between anandamide and glutamate in the control group post exercise but not in the chronic pain group. Moreover, the glutamate/anandamide ratio increased significantly in the control group and differed significantly with the chronic pain group post exercise. The altered relationship between anandamide and glutamate after the intervention in the chronic pain group might reflect alterations in the endocannabinoid-glutamate mechanistic links in the chronic pain group compared to the pain-free control group.

## Introduction

Chronic musculoskeletal pain is associated with disability, impaired quality of life and substantial socioeconomic costs.^1^ Although a mechanistic approach to address chronic pain conditions has been actively promoted for some decades, the knowledge of the biochemistry behind the emergence and maintenance of chronic musculoskeletal pain are far from complete, a situation that constrains the development of mechanism-based therapies for these conditions.^2^

Physical activity and exercise are interventions associated with few adverse events that may improve pain severity, physical function and consequently the quality of life of people suffering from chronic pain.^3^ The biochemical mechanisms behind the reduced pain due to physical exercise are not fully understood. However, exercise-induced hypoalgesia (EIH) refers to a phenomenon that describes one form of endogenous pain modulation. EIH has been characterised by elevations in pain thresholds as well as reductions in pain intensity ratings during and following exercise and has been demonstrated to activate endogenous systems including the opioid and the cannabinoid systems.^4^

In the endogenous cannabinoid system, the most well-studied endocannabinoids – (eCBs) arachidonoylethanolamide (AEA) (also known as anandamide) and 2-arachidonoylglycerol (2-AG) – activate the cannabinoid 1 and 2 (CB_1_ and CB_2_) receptors.^5,6^ Both anandamide and 2-AG have been found to have an affinity for the transient receptor potential vanilloid 1 (TRPV_1_) receptor^7,8^ and the peroxisome proliferator activating receptor (PPAR)-γ and PPAR-α.^9,10^ The *N*-acylethanolamines (NAEs) (not defined as eCBs since they lack affinity for CB receptors), oleoylethanolamide (OEA) and palmitoylethanolamide (PEA) are PPAR-α agonist,^11,12^ and OEA is also a TRPV_1_ activator.^13^ For stearoylethanolamide (SEA), no receptor target has been clearly identified, but it has been proposed to activate PPAR-γ.^14^

Multiple functions are suggested for eCBs and NAEs, including modulation of pain and inflammation.^15^ Moreover, physical exercise seems to affect circulating eCBs and NAEs in animals and humans.^16–20^

Glutamate is the primary excitatory neurotransmitter in the nervous system, affecting several ionotropic (NMDA, kaninate, AMPA) and metabotropic (mGluRs^1–8^) receptors.^21^ Accumulating evidence has demonstrated that glutamatergic signalling plays a pivotal role in pain transmission and sensation.^22^

A large amount of data supports the existence of a link between the endocannabinoid system and glutamatergic pathways in the brain. Both retrograde and non-retrograde EC signalling has been suggested to be dependent on mGluRs,^23^ and neuronal glutamate transporters play a key role in regulating endocannabinoid-mediated crosstalk between glutamatergic and GABAergic synapses within the periaqueductual gray (PAG).^24^ Furthermore, activation of TRPV_1_ inhibits glutamate release in the hippocampus of rats,^25^ and TRPV_1_ stimulation has been suggested to produce analgesia by releasing glutamate in PAG neurons.^26^ Moreover, PEA has been reported to inhibit glutamate release in rat cerebrocortical nerve terminals.^27^

Previously, we compared alterations of circulating levels of eCBs and NAEs^28,29^ and glutamate^30^ in subjects with chronic widespread pain and healthy controls (HC); however, no clear biological roles of this alteration were determined. Here, we investigate the effect of a 30-min dynamic load arm cycling intervention with respect to circulating levels of eCBs, NAEs and glutamate in subjects with chronic neck and shoulder pain (CNSP) and in HC.

## Subjects and methods

### Subjects

This study includes 21 subjects (16 women and 5 men) with CNSP and 11 HC (6 women and 5 men) without pain. The subjects with CNSP were recruited from physiotherapy clinics and the Occupational and Environmental Medicine clinic at Sahlgrenska University Hospital, Gothenburg, Sweden. The HC subjects were recruited by advertising on official message boards at the University of Gothenburg. All subjects were between 18 and 65 years old. The HC participants were required to be working and/or studying, while the subjects in the pain group could be working, studying or sick-listed.

Inclusion criteria for the chronic pain subjects were musculoskeletal pain for at least three months in neck/shoulder. Exclusion criteria were symptoms of joint involvement or tendinitis in the shoulder joint, rheumatic or metabolic disease, neurological disease, traumatically induced neck pain (whiplash), fibromyalgia diagnosis or severe mental disorder.

Exclusion criteria for the HC were neck/shoulder pain for more than two to three days during the last 12 months or severe mental disorder. The study was approved by the Regional Ethical Review Board in Gothenburg (Dnr 956–11) and was conducted according to the Helsinki Declaration.

### The arm cycling intervention

**Figure 1.**
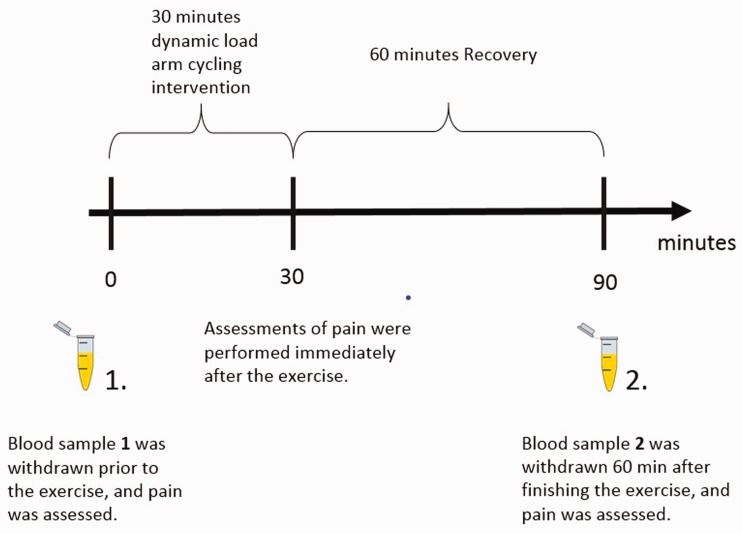
Blood sample was drawn just before the arm cycling intervention and 60 min after the intervention.

The physical exercise intervention was performed on an ergometer (Monark Cardio Rehab 891E, Monark Exercise AB, Vansbro, Sweden) so as to provoke the affected neck/shoulder muscle region. For all participants, the arm cycling started at 9 a.m. and lasted 30 min. The participants were required to maintain a steady pace of 25 laps/min. Women started with a 100 g load and the men with a 200 g load. These loads were increased to 300 g and 400 g, respectively, for woman and men, after 10 min and further increased to 500 g and 600 g, respectively, after 20 min, which was maintained for 10 min. Blood samples were collected before the intervention and 60 min after the intervention. Pain scorings were recorded before the exercise immediately after the intervention and 60 min after the intervention. Figure 1 illustrates the workflow of the arm cycling intervention, blood sampling and pain assessments.

### Pain assessments

Pain intensity and sensitivity were assessed just before (pre), immediately after (post 1) and 60 min after (post 2) the intervention. The participants were asked to rate their pain intensity on a numeric rating scale (NRS) (0–10) and with written descriptors at the two end points (0 = no pain and 10 = worst possible pain).

Pain sensitivity was assessed with pressure pain thresholds of the right and left trapezius muscles using a handheld electronic pressure algometer (Somedic, Hörby, Sweden). Some of the results concerning pain intensity have been published elsewhere,^31^ although not with the same number of subjects and time points. The results concerning sensitivity will be reported elsewhere.

### Analysis of lipid concentrations

The lipid concentrations were analysed in a blinded fashion using a liquid chromatography tandem mass spectrometry (LC-MS/MS) method based on a previously published method.^32^ Before the measurements, lipids were extracted from plasma following a previously described protocol.^29^ Briefly, 300 μL of plasma were thawed and vortexed, and 30 μL of a mixture containing the deuterated internal standard (AEA-d4, OEA-d4, PEA-d4 and SEA-d3 (50 nM)) and 2AG-d5 (1000 nM) were added to each plasma sample. C8 Octyl SPE columns (6 mL, 200 mg) (Biotage, Uppsala, Sweden) were used for lipid extraction as previously described.^29^ On the day of analysis, samples were reconstituted in 30 μL of LC mobile phase A. The injection volume was 10 μL. All standards and internal standards were purchased from Cayman Chemicals (Ann Arbor, MI, USA). We used an HPLC-MS/MS system containing a Thermo Scientific Accela AS auto sampler and Accela 1250 pump coupled to a Thermo Scientific TSQ Quantum Access max triple quadrupole mass spectrometer with an HESI II probe as an ionisation source. LC was performed using gradient elution with mobile phase A containing methanol-milliQ water-acetonitrile (4/4/2) (v/v/v) and mobile phase B containing methanol-ACN (7/3) (v/v) with 0.1% (v/v) formic acid and 1 g/L ammonium acetate in A and B. Gradient elution was applied with a constant flow of 250 μL/min. We started with 100% A during the first 1.5 min and followed this using a linear increase towards 100% B, which was achieved after 9 min in total. Between the 11th and 12th min, the gradient changed linear to 100% A, which was maintained for 1 min. An Xbridge C8 analytical column (2.1 mm × 150 mm) with the particle size 2.5 µm was obtained from Waters (Dublin, Ireland). We used the following selected reaction monitoring (m/z) transitions: 348.3/62.4, 326.3/62.4, 300.3/62.4, 328.3/62.4 and 379.3/287.3 for AEA, OEA, PEA, SEA and 2-AG, respectively. For the corresponding internal standards, we used the following transitions: 352.3/62.4, 330.3/62.4, 304.3/62.4, 331.3/62.4 and 384.3/287.3 for AEA-d4, OEA-d4, PEA-d4, SEA-d3 and 2-AG-d5, respectively. The linearity of the measuring ranges was assessed with standard curves ranging from 0.5–25 nM for AEA; 5–250 nM for OEA, PEA and SEA; and 30–1250 nM for 2-AG in duplicate. The linearity of the standard curves was R^2^ ≥ 0.9 for all analytes. Isotopic dilution was used for quantification of the analytes, which was performed according to their area ratio of their corresponding deuterated internal standard signal area. Linear regression and X^2^ weighting were applied. Undetected levels were considered as 0 nM. Xcalibur® (version 2.1, Thermo Scientific) software was used for peak integration and quantification.

### Analysis of glutamate concentrations

Concentrations of glutamate were determined according to Kreiner and Galbo.^33^ Briefly, 50 µL plasma was thawed and centrifuged at 4°C for 5 min at 12,000 r/min. The supernatant was collected and transferred to a new tube, and 10 µL was immediately analysed using a CMA ISCUSS_flex_ analyser.

### Statistics

Data analyses were performed using the IBM SPSS version 22.0 (IBM Corporation, Route 100 Somers, New York, NY, USA) and the GraphPad Prism computer programmer version 6.03 (GraphPad Software Inc., San Diego, CA, USA). Comparison of lipids and glutamate levels and pain measurements pre and post exercise between the pain group and the control group was done using repeated measures analysis of variance, resulting in a marginal model. This regression model included time points (pre and post), group (CNSP/HC) and the interaction time × group. Correlations were tested using bivariate correlation analyses (Pearson or Spearman). A *P* ≤ 0.05 was used as level of significance in all statistical analyses.

## Results

### Background data

Age expressed as mean ± standard deviation (SD) (CNSP = 50.8 ± 12.9: HC= 37.7 ± 15.9; *P *=* *0.017) was significantly higher in CNSP compared to HC. No statistically significant difference in body mass index (BMI) between groups existed (CNSP = 25.9 ± 5.5: HC = 23.2 ± 3.3; *P *=* *0.141).

### Pain scores

**Figure 2.**
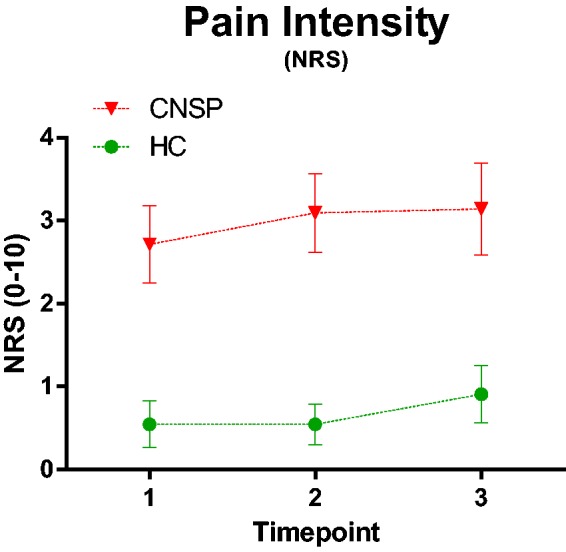
Pain intensity (expresses with numeric rating scale of pain (0–10)). Time point 1 = pre exercise, time point 2 = immediately post exercise and time point 3 = 60 min post exercise for CNSP and HC with error bars in SEM. Note that the results concerning pain intensity have partly (although not the same number of subjects and time points) been published elsewhere.^31^

Pain intensity, expressed as means ± SD (CNSP: pre = 2.7 ± 2.1; post 1 = 3.1 ± 2.1; post 2 = 3.1 ± 2.5 and HC: pre= 0.55 ± 0.93; post 1 = 0.55 ± 0.82; post 2 = 0.91 ± 1.14), was significantly higher in CNSP compared to HC (*P* < 0.01), but no statistically significant change in pain intensity as a result of the exercise existed in CNSP or HC (Figure 2). The results concerning the pain intensity scores have partly (although not the same number of subjects and time points) been published elsewhere.^31^

### Levels of lipids and glutamate before and after the intervention

No statistically significant difference was found between CNSP and HC with respect to lipid levels or glutamate before and after the arm cycling exercise. No statistically significant change was found in levels due to the arm cycling exercise except for the anandamide levels, which were increased (not statistically significant) on the group level for the pain patients and significantly decreased (*P *=* *0.036) for the HC post exercise. In CNSP, SEA levels were significantly higher (*P *=* *0.04) in women compared to men post exercise; in HC, SEA levels were significantly higher (*P *=* *0.008) in men compared to women pre exercise. Mean levels with SD of lipids and glutamate in women and men separately are presented in Supplementary Table S1.

Mean levels with statistical *P* values of lipids and glutamate for each group (CNSP and HC) pre and post exercise are presented in Tables 1 and 2.

**Table 1.** Between-group comparison of mean levels of anandamide, OEA, PEA, SEA, 2-AG (nM) and glutamate (µM) with SD for CNSP and HC pre and post 30-min arm cycling.

Compound	CNSP pre	HC pre	*P*	CNSP post	HC post	*P*
	Mean (SD)	Mean (SD)		Mean (SD)	Mean (SD)	
Anandamide (nM)	0.84 (0.09)	0.85 (0.14)	0.94	0.92 (0.09)	0.63 (0.13)	0.06
OEA (nM)	7.57 (1.93)	6.09 (2.04)	0.10	7.94 (2.2)	6.48 (1.84)	0.08
PEA (nM)	7.18 (1.68)	6.79 (2.19)	0.81	7.23 (1.32)	7.07 (1.83)	0.78
SEA (nM)	6.18 (1.31)	5.74 (1.05)	0.55	6.05 (1.75)	6.65 (3.08)	0.49
2-AG (nM)	16.5 (6.40)	22.0 (15.66)	0.13	18.9 (6.64)	23.1 (15.48)	0.30
Glutamate (µM)	47.0 (27.1)	49.7 (26.6)	0.78	42.7 (21.7)	52.3 (32.7)	0.33

Note: Statistical comparison expressed as *P*-values for CNSP versus HC pre and post exercise. CNSP: chronic neck and shoulder pain; HC: healthy controls; SD: standard deviation; OEA: oleoylethanolamide; PEA: palmitoylethanolamide; SEA: stearoylethanolamide; AG: arachidonoylglycerol.

**Table 2.** Within-group comparison of mean levels of anandamide, OEA, PEA, SEA, 2-AG (nM) and glutamate (µM) with SD for CNSP and HC pre and post 30-min arm cycling exercise.

Compound	CNSP pre	CNSP post	*P*	HC pre	HC post	*P*
	Mean (SD)	Mean (SD)		Mean (SD)	Mean (SD)	
Anandamide (nM)	0.84 (0.09)	0.92 (0.09)	0.25	0.85 (0.14)	0.63 (0.13)	0.04^a^
OEA (nM)	7.57 (1.93)	7.94 (2.2)	0.26	6.09 (2.04)	6.48 (1.84)	0.65
PEA (nM)	7.18 (1.68)	7.23 (1.32)	0.85	6.79 (2.19)	7.07 (1.83)	0.87
SEA (nM)	6.18 (1.31)	6.05 (1.75)	0.78	5.74 (1.05)	6.65 (3.08)	0.30
2-AG (nM)	16.5 (6.40)	18.9 (6.64)	0.28	22.0 (15.66)	23.1 (15.48)	0.93
Glutamate (µM)	47.0 (27.1)	42.7 (21.7)	0.31	49.7 (26.6)	52.3 (32.7)	0.66

Note: Statistical comparison expressed with *P*-values for CNSP and HC pre versus post exercise. CNSP: chronic neck and shoulder pain; HC: healthy controls; SD: standard deviation; OEA: oleoylethanolamide; PEA: palmitoylethanolamide; SEA: stearoylethanolamide; AG: arachidonoylglycerol.

^a^Statistical significant difference.

### Bivariate correlations

No significant correlation existed between age and levels of lipids or glutamate at baseline in the two groups. BMI correlated positively with PEA (r = 0.81, 95% confidence interval (CI): 0.403–0.948) and 2-AG (r = 0.65, 95% CI: 0.079–0.899) in HC at baseline.

No significant correlations between pain scores and levels of the investigated molecules existed in the two groups (CNSP and HC) before or after the intervention.

In HC, anandamide correlated positively with OEA pre exercise (r = 0.81, 95% CI: 0.405–0.948) and post exercise (r = 0.87, 95% CI: 0.524–0.968) and with PEA (r = 0.80, 95% CI: 0.390–0.946) and post exercise (r = 0.81, 95% CI: 0.357–0.952).

In CNSP, anandamide correlated positively with OEA pre (r = 0.81, 95% CI: 0.589–0.921) and post (r = 0.78, 95% CI: 0.529–0.907) exercise. Anandamide and PEA correlated positively post exercise (r = 0.66, 95% CI: 0.311–0.847).

**Figure 3.**
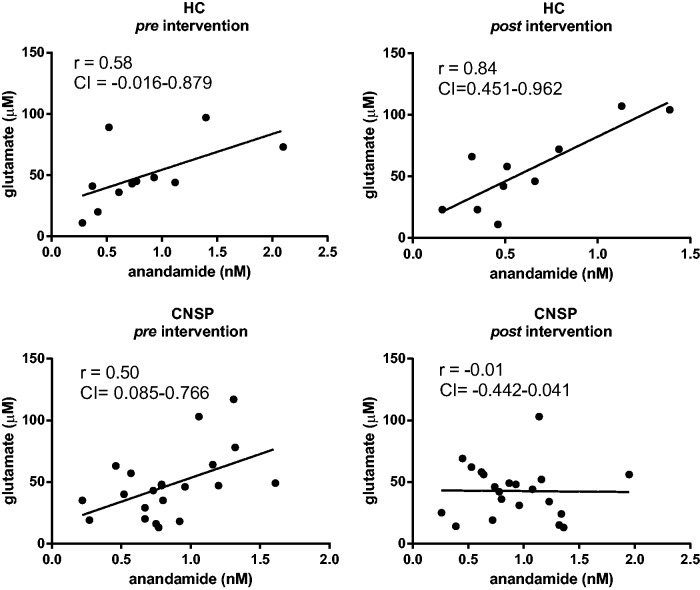
Scatterplots of anandamide and glutamate in HC (in the two upper panels) and in CNSP (in the two lower panels) pre and post 30-min arm cycling intervention, including Pearson’s *r* and 95% confidence interval.

Significant positive correlations existed between anandamide and glutamate in HC post exercise (r = 0.84, 95% CI: 0.451–0.962) and in CNSP pre exercise (r = 0.50, 95% CI: 0.085–0.766). Correlation plots of anandamide–glutamate from HC and CNSP pre and post exercise are shown in Figure 3.

### Relationship between anandamide and glutamate – An explorative ratio analysis

**Figure 4.**
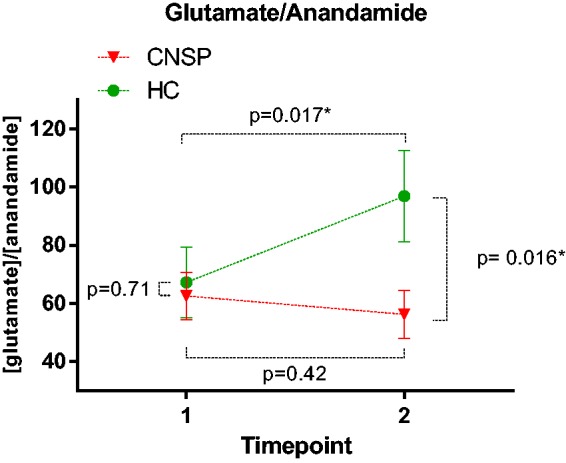
Ratio levels with error bars in SEM of glutamate and anandamide in chronic neck and shoulder pain and healthy controls before and after (time points 1 and 2) the 30-min arm cycling intervention, with *P*-values where asterisk (*) indicates statistical significant difference.

To further evaluate the relationship between anandamide and glutamate in circulation, their ratio was calculated in the two groups –pre and post the intervention. Interestingly, a statistically significant increase in glutamate/anandamide ratio existed in HC. The following mean ratios (SD in bracket) were calculated for the HC: pre = 67.3 (40.2) and post = 96.9 (49.6) (*P* = 0.017). The following mean ratios (SD in bracket; non-significant) were calculated for the CNSP, which were non-significant decrease: pre = 62.6 (37.2) and post = 56.2 (37.3). A significant difference existed between CNSP and HC post exercise (*P* = 0.016) (Figure 4).

## Discussion

The main finding in this study was that anandamide and glutamate were positively correlated in a similar manner before the intervention in CNSP and HC. After 30 min arm cycling, this positive correlation became substantially stronger in HC but was lost in CNSP.

According to the literature, the quality and quantity of a physical exercise required to reach a measurable change in eCBs and NAEs levels seems to be important. Raichlen et al. reported that circulating anandamide levels are significantly increased in response to 30 min of running, but not to 30 min of walking in humans (n = 10) and dogs (n = 8).^19^ NAEs increased significantly in healthy trained male cyclists (n = 11) immediately after 60 min of cycle exercise and continued to increase after 15 min of recovery.^20^ Cedernaes et al. found 2-AG levels to significantly increase 15 min after finishing 30 min of cycling on an ergometer in 16 healthy males; however, no effect on anandamide levels was found in response to this intervention.^34^ The 30 min of arm cycling intervention in this study did not result in any significant change in eCB/NAE levels except for anandamide levels in the HC group, which were statistically significantly decreased post the intervention. This result could most likely be explained by the type of intervention used in this study, which in comparison with the above reports was a relatively low-force intervention. Moreover, since blood samples were drawn 60 min after the completion of the intervention in our study and not immediately after intervention, the immediate effect of the physical activity on lipid and glutamate levels was not revealed. These circumstances further aggravate the comparison with other reports.

Acute exercise might be influenced by biochemical components (in periphery and/or in brain): lactate, cortisol, brain derived neurotropic factor, insulin-like growth factor-1, vascular endothelial growth factor, dopamine, norepinephrine, serotonin, acetylcholine, GABA, glutamate and endogenous opioids and cannabinoids.^35^ However, studies investigating acute physical activity/exercise in chronic pain subjects and endogenous chemistry are relatively limited. In one study, changed levels of pro-inflammatory cytokines in microdialysate sampled from vastus lateralis muscles were reported after a 20-min leg muscle work intervention in subjects with fibromyalgia (n = 32) and in HC (n = 32).^36^ In another study, significantly decreased levels of PEA and SEA sampled from the trapezius muscle in subjects with chronic widespread pain (n = 18) after a 20-min standardised low-force repetitive exercise were reported, but no level changes were evident in CNSP subjects (n = 34) or in HC (n = 24) after the brief work.^37^

The link between the ECs and glutamatergic signalling was revealed almost two decades ago when CB_1_ receptor agonists was discovered to dampen glutamatergic transmission,^38^ and blockade of CB_1_ was found to protect against NMDA-induced excitotoxicity in rat brains.^39^ In addition to the NMDA interaction, CB_1_ also crosstalk with mGlu_5_. The mGlu_5_/CB_1_ signalosome complex might play a major role in nociception.^40^ The literature has very little to say about the link between the ECs and glutamatergic pathways in the periphery and the importance of the relationship between circulating levels of mediators of the EC systems and glutamate. To the best of our knowledge, this is the first study to report about the relationship of circulating anandamide and glutamate levels in chronic pain patients and HC before and after a physical exercise intervention.

If the increased correlation between anandamide and glutamate (Figure 3) in HC after the arm cycling reflect normal function, the decreased correlation between these compounds (Figure 3) in the chronic pain group might reflect abnormal function, which in turn could be reflecting alterations in the interplay between the ECs and glutamatergic pathways at some level. The altered anandamide–glutamate relation between CNSP and HC after the intervention is also illustrated clearly in the exploratory ratio analysis (Figure 4).

Concerning the origin of blood levels of endocannabinoids, the available data suggest that brain concentrations are not related to blood concentrations in a direct manner, they are instead suggested to originate from different organs and tissues, including brain, muscle, adipose tissue and circulating cells as an indirect marker of tissue endocannabinoid tone.^41^ Concerning the origin of circulating glutamate, serum and cerebrospinal fluid levels of glutamate were reported to correlate positively in healthy volunteers.^42^ Glutamate blood and brain levels were also reported to be highly correlated in children with autistic disorder,^43^ but no association between plasma and brain glutamate levels was found in healthy males,^44^ suggesting that peripheral and central levels of glutamate are associated under certain circumstances but not intrinsically.

Circulating eCBs and NAEs levels have been reported to be influenced by age, BMI, diet and food consumption. Age has been reported to positively influence 2-AG and PEA in Fanelli et al.,^45^ and BMI and circulating 2-AG have been reported to be positively correlated. This study found positive correlations between BMI, PEA and 2-AG in the HC group. Moreover, OEA, PEA and SEA have been reported to be influenced by diet,^46,47^ and circulating OEA increases in humans after consuming a diet enriched in monounsaturated fat.^48^ Diet was not controlled for in this study, which is a limitation. Another limitation (as described above) was that blood samples were drawn 1 h after the intervention rather than immediately after the intervention.

## Conclusions

The 30-min arm cycling intervention affected the relationship between plasma levels of anandamide and glutamate significantly different in the chronic pain group compared to the pain-free group. This difference could indicate that links between the ECs and glutamatergic pathways are altered in chronic pain. Moreover, the ratio of glutamate/anandamide in plasma could be an important marker when investigating the effects of physical exercise in chronic pain.

## Supplemental Material

MPX898360 Supplemental Material - Supplemental material for Altered relationship between anandamide and glutamate in circulation after 30 min of arm cycling: A comparison of chronic pain subject with healthy controls Click here for additional data file. Supplemental material, MPX898360 Supplemental Material for Altered relationship between anandamide and glutamate in circulation after 30 min of arm cycling: A comparison of chronic pain subject with healthy controls by Niclas Stensson and Anna Grimby-Ekman in Molecular Pain
