# Supplementary material for: Altered relationship between anandamide and glutamate in circulation after 30 min of arm cycling: A comparison of chronic pain subject with healthy controls
Source: Mol Pain. 2019 Dec 30;15:1744806919898360. doi: 10.1177/1744806919898360 (PMC6964246; doi:10.1177/1744806919898360)
Supplement: MPX898360 Supplemental Material - Supplemental material for Altered relationship between anandamide and glutamate in circulation after 30 min of arm cycling: A comparison of chronic pain subject with healthy controls [file MPX898360_Supplemental_Material.pdf]

Table S1. Mean levels (with SD in brackets) of arachidonoylethanolamide (anandamide), oleoylethanolamide (OEA), palmitoylethanolamide (PEA), stearoylethanolamide, (SEA) 2-aracidonoylglycerol, (2-AG) (nM), and glutamate (μM) in women and men from the healthy controls (HC) and from the chronic neck and shoulder pain (CNSP) group.

**Compound**	***AEA** (nM)*	***OEA** (nM)*	***PEA** (nM)*	***SEA** (nM)*	***2-AG** (nM)*	***GLT** (μM)*
HC women pre (n=6)	0.78 (0.41)	5.50 (1.82)	5.86 (1.62)	5.06 (0.54)	14.66 (3.43)	53.7 (32.9)
HC men pre (n=5)	0.91 (0.70)	6.80 (2.28)	6.56 (0.91)	6.56 (0.91)	30.83 (20.49)	45.0 (18.7)
HC women post (n=6)	0.71 (0.27)	6.33(1.42)	7.09 (1.45)	7.71 (3.81)	17.92 (9.23)	52.8 (34.6)
HC men post (n=5)	0.54 (0.49)	6.62 (2.36)	7.07 (2.34)	5.58 (2.02)	28.25 (19.67)	51.6 (34.2)
CNSP women pre (n=16)	0.84 (0.37)	7.69 (2.00)	7.02 (1.73)	6.25 (1.48)	15.86 (6.66)	43.6 (25.9)
CNSP men pre (n=5)	0.82 (0.32)	7.18 (1.82)	7.72 (1.52)	5.96 (0.56)	18.34 (5.69)	57.6 (31.7)
CNSP women post (n=16)	0.91 (0.44)	8.25 (2.42)	7.22 (1.47)	6.31 (1.93)	17.43 (6.03)	40.1 (17.6)
CNSP men post n= 5)	0.93 (0.29)	6.95 (0.81)	7.31 (0.78)	5.20 (0.22)	23.47 (7.04)	50.8 (33.0)
